# Syndrome de Gougerot-Sjögren primitif du sujet âgé: caractéristiques cliniques et immunologiques

**DOI:** 10.11604/pamj.2015.20.8.5042

**Published:** 2015-01-05

**Authors:** Wafa Chebbi, Wafa Ben Salem, Rim Klii, Wassia Kessomtini, Saida Jerbi, Mohamed Habib Sfar

**Affiliations:** 1 Service de Médecine Interne, CHU Taher Sfar, Mahdia, Tunisie; 2 Service de Médecine Interne, CHU Fattouma Bourguiba, Monastir, Tunisie; 3 Unité de Médecine Physique, CHU Taher Sfar Mahdia, 5100 Mahdia, Tunisie; 4 Service de Radiologie, CHU Taher Sfar, Mahdia, Tunisie

**Keywords:** Syndrome de Gougerot-Sjögren primitif, personnes âgées, Primitive Sjögren syndrome, elderly

## Abstract

Les objectifs de notre étude est d’étudier les caractéristiques cliniques et immunologiques du syndrome de Gougerot Sjogren primitif (SGSp) du sujet âgé et les comparer à une population témoin plus jeune. Nous avons analysé rétrospectivement les dossiers de 60 patients atteints de SGSp consécutifs, suivis au service de Médecine interne du CHU Taher Sfar de Mahdia, en Tunisie, durant une période de 7 ans (janvier 2007 à décembre 2013). Les patients avec SGSp étaient classés en deux groupes selon l’âge de début de la maladie: groupe de sujets âgés (≥ 65 ans) et groupe de sujets jeunes (<65 ans). Chez 18 patients (30%), le SGSp a débuté après 65 ans. Il s'agissait de 17 femmes (94,5%) et un homme (5,5%). L’âge moyen du début du SGSp était de 70,5 ans ±3,1. Le délai moyen du diagnostic était de 38,6 mois ±26,8. Les manifestations glandulaires étaient constantes. La comparaison entre le groupe du SGSp du sujet âgé et celui du sujet jeune montrait que le délai moyen du diagnostic du SGSp était significativement plus long chez les sujets âgés. Les manifestations pulmonaires étaient significativement plus fréquentes chez les sujets âgés. En revanche, les fréquences des anticorps antinucléaires, anti-SSA et anti-SSB étaient significativement plus élevées chez les sujets jeunes. Bien que le SGSp soit une maladie typique d'adultes d’âge moyen, les cliniciens ne devraient pas ignorer que cette maladie peut être diagnostiquée aussi chez les patients âgés. Notre étude montre que l’âge du début avancé influence le profil clinique et immunologique du SGSp.

## Introduction

Le syndrome de Gougerot-Sjögren (SGS) est une maladie inflammatoire auto-immune caractérisée par un tarissement des larmes, responsable d'une xérophtalmie et la disparition de la sécrétion salivaire entrainant une xérostomie. Le support histologique de cette pathologie est une infiltration lymphocytaire qui concerne principalement le tissu glandulaire exocrine, mais qui peut être aussi extraglandulaire. Sa physiopathologie reste imparfaitement élucidée, faisant intervenir des facteurs génétiques, environnementaux et immunologiques [1]. Le spectre du SGS s’étend d'un désordre auto-immun organo-spécifique (exocrinopathie auto-immune) à un processus systémique qui peut atteindre les systèmes musculo-squelettique, pulmonaire, hématologique, vasculaire, cutané, rénal ou nerveux [1–3]. Le SGS peut être primitif ou secondaire associé à d'autres maladies auto-immunes.

Le syndrome de Gougerot-Sjögren primitif (SGSp) affecte 0,3 à 5% de la population. Il peut survenir à tout âge, mais touche préférentiellement les femmes entre la quarantaine et la cinquantaine, avec un sex ratio femme/homme de 9/1 ou même plus [3]. Dans la littérature, plusieurs études ont démontré que l’âge de début influence l'expression clinico-biologique, le mode évolutif ainsi que le pronostic des pathologies auto-immunes, telles que le lupus érythémateux systémique [4, 5] et la polyarthrite rhumatoïde [6]. A notre connaissance, seules peu d’études ont été publiées sur les caractéristiques cliniques et immunologiques du SGSp chez le sujet âgé et dont les résultats sont discordants [7–10] et à ce jour, il n'existe aucune étude Nord Africaine faite dans ce sens. L'objectif de ce travail est de déterminer, à travers une étude rétrospective et comparative, les caractéristiques cliniques et immunologiques des patients dont l’âge de début des premières manifestations imputables au SGSp était supérieur ou égal à 65 ans et de les comparer à une population témoin plus jeune.

## Méthodes

### Population d’étude

Nous avons analysé rétrospectivement les dossiers de 60 patients atteints de SGSp consécutifs, suivis au service de Médecine interne du CHU Taher Sfar de Mahdia, en Tunisie, durant une période de 7 ans s’étalant du janvier 2007 à décembre 2013. Tous les patients satisfaisaient à 4 ou plus des critères élaborés par le groupe de consensus américano-européen révisés en 2002 [11]. Aucun de ces patients ne présentait de preuve clinique ou immunologique d'une autre maladie auto-immune. L’âge de début est défini par l'apparition de premières manifestations fortement évocatrices du SGSp. Les patients étaient classés en deux groupes selon l’âge de début du SGSp: groupe de sujets âgés (≥ 65 ans) et groupe de sujets jeunes (<65 ans).

### Recueil des données

Les données ont été répertoriées sur une fiche d'exploitation incluant l’âge, le sexe, l’âge de d'apparition des premiers symptômes, l’âge au moment du diagnostic, le délai du diagnostic (durée qui sépare la date d'apparition du premier signe de la maladie de la date du diagnostic définitif), les circonstances de découverte, le mode de début, les manifestations cliniques, les résultats du test de Schirmer, de l’étude anatomopathologique de la biopsie des glandes salivaires accessoires, de la scintigraphie des glandes salivaires, des examens biologiques (hémogramme, vitesse de sédimentation, électrophorèse des protéines plasmatiques, créatininémie, ionogramme sanguin) et du bilan immunologique (anticorps antinucléaires (AAN), anti-SSA et anti-SSB).

### Analyse statistique

L'analyse des données statistiques a été réalisée à l'aide du logiciel SPSS 11.0. Les résultats ont été exprimés par la moyenne ± déviation standard pour les variables quantitatives et par les pourcentages pour les variables qualitatives. La comparaison des moyennes a été effectuée par Student test ou Kruskal-Wallis test. L'association entre les variables qualitatives a été réalisée par le test de Chi2 corrigé par le test exact de Fisher. Une valeur p < 0,05 a été considérée comme significative.

## Résultats

### Données épidémiologiques

Chez 18 patients (30%), le début de du SGSp est survenu après l’âge de 65 ans. Il s'agissait de 17 femmes (94,5%) et un homme (5,5%). L’âge moyen du début du SGSp était de 70,5 ans ±3,1 avec des extrêmes allant de 67 à 77 ans. Le délai moyen du diagnostic du SGSp était de 38,6 mois ±26,8 (18-60 mois).

### Manifestations cliniques

Les principales manifestations cliniques sont représentées sur le Tableau 1. La xérophtalmie et la xérostomie étaient observées chez tous les patients. Le test de Schirmer était positif dans tous les cas. Une kératoconjonctivite sèche était objectivée chez 12 patients (66,6%). Six patients (33,3%) présentaient une hypertrophie parotidienne bilatérale récidivante. La biopsie des glandes salivaires accessoires a mis en évidence un stade III ou IV de la classification de Chisholm dans 88,8% des cas. La scintigraphie des glandes salivaires, pratiquée chez 16 patients, objectivait une captation faible des glandes salivaires dans 15 cas (93,7%). Les manifestations extraglandulaires étaient observées chez 17 patients (94,5%). L'atteinte articulaire représentait la manifestation systémique la plus fréquente observée chez 16 patients (88,8%). Il s'agissait des arthralgies de type inflammatoire chez 15 patients et une polyarthrite symétrique non destructive chez une seule patiente. Sept patients (38,8%) avaient présenté des manifestations neuropsychiatriques. Les atteintes du système nerveux périphérique étaient les plus fréquentes, observées chez 6 patients (33,3%). Il s'agissait d'une polyneuropathie axonale sensitive dans 5 cas et une mononeuropathie multiple sensitivomotrice sévère des nerfs médians et sciatiques poplités externes dans un cas. Une atteinte du système nerveux central était observée chez 5 patients (27,7%). Elle était associée à une atteinte périphérique dans 4 cas. L'atteinte pulmonaire était observée chez 8 patients (44,4%). Le scanner thoracique révélait une pneumopathie interstitielle diffuse chez 5 patients et une fibrose interstitielle diffuse dans 3 cas. L'EFR objectivait un syndrome restrictif dans les 8 cas. Une atteinte rénale à type d'acidose tubulaire distale était observée chez deux patients (11,1%). Un purpura vasculaire siégeant au niveau des membres inférieurs était constaté au moment du diagnostic du SGSp chez un seul patient. La biopsie cutanée objectivait une vascularite leucocytoclasique. Un phénomène de Raynaud survenant au cours de l’évolution du SGSp était observé chez une seule patiente. La capillaroscopie montrait des signes de microangiopathies organiques. Des adénopathies cervicales étaient observées dans un seul cas et dont la biopsie montrait une lymphadénite réactionnelle non spécifique.

**Tableau 1:** Comparaison des caractéristiques épidémiologiques et cliniques du syndrome du Gougerot Sjogren primitif du sujet âgé et du sujet jeune

Paramètres	Sujets âgés n=18	sujets adultes n=42	P
**Caractéristiques démographiques**			
Femmes n (%)	17 (94,5%)	41 (97,6%)	NS
Age de début (ans)	70,5±3,1	46,2±10,1	
Age au moment du diagnostic (ans)	73,6±2,9	47,5±10,1	
Délai du diagnostic (mois)	38,6±26,8	17,7±12,4	0,005
**Manifestations cliniques**			
Atteinte glandulaire	18 (100%)	42 (100%)	NS
Atteinte articulaire	16 (88,8%)	35 (83,3%)	NS
Atteinte neurologique	7 (38,8%)	10 (23,8%)	NS
Atteinte pulmonaire	8 (44,4%)	6 (14,2%)	0,019
Atteinte rénale	2 (11,1%)	1 (2,4%)	NS
Purpura vasculaire	1 (5,5%)	1 (2,4%)	NS
Syndrome de Raynaud	1 (5,5%)	2 (4,7%)	NS
Adénopathie	1 (5,5%)	1 (2,4%)	NS

### Données biologiques

Les données biologiques des patients sont résumées dans le Tableau 2. Une anémie était observée dans 7 cas (38,8%). Trois patients (16,6%) avaient une leucopénie. Une thrombopénie était constatée dans un seul cas (5,5%). Un SIB était noté dans 12 cas (66,6%). Une élévation de la vitesse de sédimentation (VS) était constatée chez 15 patients (83,3%) avec une valeur moyenne de 70,4 ± 6,1 mm à la première heure. La VS était supérieure à 100 mm à la première heure dans 4 cas. Une hypergammaglobulinémie polyclonale était constatée dans 16 cas (88,8%). La recherche des AAN était positive chez 8 patients (44,4%). Les anticorps anti-SSA et anti-SSB étaient positifs respectivement dans 33,3% et 16,6%.

**Tableau 2:** Comparaison des caractéristiques biologiques du syndrome du Gougerot Sjogren primitif du sujet âgé et du sujet jeune

Paramètres	Sujets âgés n=18	sujets jeunes n=42	P
Anémie	7 (38,8%)	8 (19%)	NS
Leucopénie	3 (16,6%)	3 (7,1%)	NS
Thrombopénie	1 (5,5%)	2 (4,7%)	NS
Syndrome inflammatoire biologique	12 (66,6%)	17 (40,4%)	NS
Hypergammaglobulinémie polyclonale	16 (88,8%)	35 (83,3%)	NS
Anticorps antinucléaires positifs	8 (44,4%)	34 (80,9%)	0,005
Anticorps anti-SSA positifs	6 (33,3%)	29 (69%)	0,01
Anticorps anti-SSB positifs	3 (16,6%)	19 (45,2%)	0,035

### Etude comparative

La comparaison des caractéristiques cliniques et biologiques entre le groupe du SGSp du sujet âgé et celui du sujet jeune (Tableau 1, Tableau 2) montrait que le délai moyen du diagnostic du SGSp était significativement plus long chez les sujets âgés (38,6 mois ± 26,8 vs 17,7 mois ± 12,4, p=0,04). Les manifestations pulmonaires étaient significativement plus fréquentes chez les sujets âgés. En revanche, les fréquences des AAN, des anticorps anti-SSA et anti-SSB étaient significativement plus élevées chez les sujets jeunes.

## Discussion

Notre étude a montré qu'un début du SGSp à un âge avancé (≥ 65 ans) n'est pas rare; sa fréquence par rapport au nombre total du SGSp était de 30%. Cette fréquence était plus élevée que celle rapportée dans la littérature où elle varie de 6 à 20% [7, 9, 10] (Tableau 3). Une prédominance féminine était constatée dans notre série ainsi que dans les autres études [7, 9, 10]. Toutes les études s'accordent sur un délai du diagnostic long du SGSp du sujet âgé. Il était de 38,6 mois ±26,8 dans notre étude. Ce délai était relativement court par rapport à celui retrouvé par Botsios (116,4 mois ± 40,8) [10]. Ce délai du diagnostic long peut être expliqué par la discrétion des signes fonctionnels du syndrome sec au début et par l'ubiquité des atteintes systémiques parfois trompeurs, se mêlant à d'autres pathologies.

**Tableau 3:** Caractéristiques épidémiologiques du syndrome de Gougerot Sjogren primitif du sujet âgé selon la littérature

Auteurs /Pays	Garcia-Carrasco et al. 7. (Espagne)	Tishler et al. 9. (Israël)	Botsios et al. 10. (USA)	Notre étude (Tunisie)
Nombre total du SGSp	223	85	190	60
Nombre du sujet âgé (%)	31 (14%)	17 (20%)	21 (6%)	18 (30%)
Age moyen du début (ans)	74	71	72,9±3,8	70,5 ±3,1
Sex ratio (F/M)	5,2	16	21	16
Délai du diagnostic (mois)	25	-	116,4±40,8	38,6 ±26,8

SGSp: Syndrome de Gougerot Sjogren primitif

Sur le plan clinique, les manifestations glandulaires sont fréquentes au cours du SGSp du sujet âgé avec une fréquence variant 76,1 à 100% selon les études [7, 9, 10]. Elles étaient constantes dans notre étude. Les fréquences des manifestations systémiques extraglandulaires au cours du SGSp du sujet âgés sont diversement appréciées dans la littérature (Tableau 4). Cette variabilité est probablement multifactorielle, pouvant être expliquée par le mode de recrutement (service de médecine interne, de neurologie…), par l'utilisation des critères d'inclusion différents ou par la réalisation ou non d'examens systématiques (électromyogramme, imagerie, EFR...). L'atteinte articulaire constitue l'atteinte extraglandulaire la plus fréquente au cours du SGSp du sujet âgé, avec une fréquence variant de 29 à 66,7% selon les études [7, 9, 10]. Elle était de 88,8% dans notre étude.

**Tableau 4:** Fréquences comparées des caractéristiques clinques et immunologiques du syndrome de Gougerot Sjogren primitif du sujet âgé dans la littérature

	Garcia-Carrasco et al. 7. (n=31)	Tishler et al. 9. (n=17)	Botsios et al. 10. (n=21)	Notre étude (n=18)
Xérostomie	97	100	71,4	100
Xérophtalmie	90	94	76,1	100
Hypertrophie des parotides	13	12	-	30
Atteinte articulaire	29	18	66,7	88,8
Atteinte du système nerveux	16	0	4,7	66,6
Atteinte pulmonaire	13	-	4,7	44,4
Atteinte rénale	-	-	4,7	11,1
Atteinte cutanée	7	-	14,2	5,5
Phénomène de Raynaud	7	12	23,8	5,5
Atteinte hématopoïétique	-	-	14,2	5,5
Anticorps antinucléaires	64	36	85,7	44,4
Anticorps anti-SSA positifs	17	12	66,7	33,3
Anticorps anti-SSB positifs	17	12	52,3	16,6

Les manifestations hématologiques biologiques sont fréquentes au cours du SGSp du sujet âgé. Les principales anomalies sont l'anémie et/ou la leucopénie. Elles étaient observées respectivement dans 38,8% et 16,6% dans notre étude. Dans l’étude de Botsios [10], ces anomalies étaient constatées respectivement dans 28,5% et 19%. La thrombopénie est beaucoup plus rare; elle était constatée dans 9,5% dans l’étude de Botsios et dans 5,5% dans notre étude. L'hypergammaglobulinémie polyclonale était plus fréquente dans notre étude (88,8%) par rapport à celle de Botsios [10], où elle était constatée dans 47,6%. La prévalence des AAN au cours du SGSp du sujet âgé varie de 36 à 85,7% selon les études [7, 9, 10]. Elle était de 44,4% dans notre étude. Les anticorps anti-SSA et anti-SSB sont très spécifiques du SGSp. La prévalence de ces différents anticorps à travers la littérature est illustrée dans le Tableau 4.

A travers la comparaison entre le groupe du SGSp du sujet âgé à une population témoin plus jeune, nous avons constaté que le délai moyen pour établir le diagnostic du SGSp était significativement plus long chez les sujets âgés. Les manifestations pulmonaires étaient significativement plus fréquentes chez les sujets âgés. Contrairement à notre étude, ces constatations n’étaient pas confirmées dans les différentes études de la littérature [7–10], où le délai moyen du diagnostic et l'expression clinique du SGS chez les patients âgés étaient similaires à ceux du sujet jeune. Dans les études de Garcia-Carrasco [7] et Botsios [10], l'expression immunologique du SGS chez les patients âgés était similaire à celle du sujet jeune. Contrairement à ces études, Haga et al. [8] ont trouvé une influence significative de l’âge sur les anomalies sérologiques du SGSp avec des fréquences plus élevées des anticorps anti-SSA et anti-SSB chez le sujet jeune. Tishler et al [9] ont observé également une fréquence signicativement élevée d'anticorps anti-SSA chez le suet jeune. Ces constatations étaient similaires à notre étude, où les fréquences des AAN, des anticorps anti-SSA et anti-SSB étaient significativement plus élevées chez les sujets jeunes. Bien que l'explication de cette variabilité dans l'expression immunologique de la maladie liée à l’âge, soit toujours peu évidente, des facteurs épidémiologiques et des différences de prédisposition génétique ou de réactivité d'un système immunitaire vieillissant pourraient être impliqués [12]. Il a été suggéré que les patients âgés et les patients jeunes atteints de maladies auto-immunes systémiques pourraient présenter des déterminants génétiques différents et répondre à des mécanismes déclencheurs différents [13–15]. L'expression moins excessive des caractéristiques immunologiques au cours de SGSp des sujets âgés peut refléter la sénescence du système immunitaire [16].

## Conclusion

Bien que le SGSp soit une maladie typique d'adultes d’âge moyen, les cliniciens ne devraient pas ignorer que cette maladie peut être diagnostiquée aussi chez les patients âgés. Dans notre étude, l'expression clinique et immunologique du SGSp chez les patients âgés était différente de celle du sujet jeune. Des cohortes plus larges sont nécessaires, afin de déterminer les caractéristiques clinques, immunologiques et évolutives du SGSp du sujet âgé.
